# Sexually transmitted infection prevalence and *Neisseria gonorrhoeae* antimicrobial resistance patterns in men who have sex with men with or without urethral discharge syndrome in Johannesburg, South Africa, 2024

**DOI:** 10.1371/journal.pgph.0006256

**Published:** 2026-04-17

**Authors:** Etienne E. Müller, Mpumelelo Sibanda, Mahlape P. Mahlangu, Johanna M. E. Venter, Lindy Y.E. Gumede, Duduzile Valashiya, Dumisile V. Maseko, Frans Radebe, Thabitha Mathega, Portia Baloyi, Nelisiwe Swana, Tendesayi Kufa, Maurice Greeves, Joseph Adams, Magnus Unemo, Ismail Maatouk, Bianca Da Costa Dias

**Affiliations:** 1 Centre for HIV and Sexually Transmitted Infections, National Institute for Communicable Diseases, National Health Laboratory Service, Johannesburg, South Africa; 2 Engage Men’s Health, Johannesburg, South Africa; 3 School of Public Health, Faculty of Health Sciences, University of the Witwatersrand, Johannesburg, South Africa; 4 WHO Collaborating Centre for Gonorrhoea and Other STIs, Örebro University, Örebro, Sweden; 5 Institute for Global Health, University College London (UCL), London, United Kingdom; 6 Global HIV, Hepatitis and STI Programmes, World Health Organization (WHO), Geneva, Switzerland; PLOS: Public Library of Science, UNITED STATES OF AMERICA

## Abstract

We conducted a cross-sectional study to estimate the prevalence of urogenital and extragenital sexually transmitted infections (STIs) among men who have sex with men (MSM) with and without urethral discharge syndrome, to assess infections missed by syndromic management, and to describe phenotypic antimicrobial susceptibility patterns of *Neisseria gonorrhoeae*. The study included 189 MSM attending the Engage Men’s Health Clinic in Johannesburg, South Africa, in 2024. Urethral, rectal and oropharyngeal swabs were tested by multiplex PCR for *Neisseria gonorrhoeae* (NG), *Chlamydia trachomatis* (CT), *Mycoplasma genitalium* (MG) and *Trichomonas vaginalis* (TV). Genital ulcer swabs were tested for HSV-1/2, lymphogranuloma venereum (LGV), *Haemophilus ducreyi* (HD) and *Treponema pallidum* (TP). Serological diagnostic testing for HIV, hepatitis B virus (HBV) and syphilis was performed. NG isolates underwent culture and antimicrobial susceptibility testing. Among MSM with urethritis, NG was most prevalent (urethra: 80.3%, rectum: 41.7%), followed by CT (urethra: 14.8%, rectum: 11.7%). Pharyngeal NG was more common among MSM with urethritis than those without (18.0% vs 3.1%, p = 0.001). Among MSM without urethritis, rectal NG and CT prevalence were 13.5% and 9.5%; rectal LGV was detected in three cases. Among 22 participants with genital ulcers, an aetiology was identified in eight: HSV-2 (n = 3), TP (n = 4) and LGV (n = 1). All NG isolates were susceptible to ceftriaxone, cefixime, and azithromycin, and had low MICs of gentamicin. HIV, HBsAg and treponemal antibody seroprevalence were 31.4%, 3.2% and 50.8%. Active syphilis (RPR titres ≥1:32) was more frequent among MSM without urethral symptoms. Among MSM with urethritis, CT infection was less likely in those reporting recent insertive oro-anal sex and HIV pre-exposure prophylaxis (PrEP) use. Among MSM without urethritis, any discharge STI was associated with homosexual orientation, HIV positivity and lack of circumcision, NG with HIV positivity and being uncircumcised and CT with HIV positivity. Extragenital and asymptomatic STIs remain common among MSM in Johannesburg, stressing the need for routine multi-site molecular screening and inclusion of rapid serological syphilis testing in national STI guidelines for key populations.

## Introduction

In South Africa, men who have sex with men (MSM) are a key population at increased risk for acquiring and transmitting sexually transmitted infections (STIs). This increased risk is driven by high-risk sexual behaviours and limited access to sexual health services that are specifically tailored to MSM. The majority of MSM rely on the public healthcare sector for HIV and STI services, where they frequently encounter stigma, discrimination and societal pressures that discourage them from seeking care [1,2]. STIs in this population can affect both urogenital and extragenital anatomical sites, often presenting without notable symptoms in the oropharynx and anorectum and resulting in ongoing transmission [3,4]. In the public healthcare system in South Africa, STIs are managed using a syndromic approach, where antimicrobial treatment is based on the presence of urogenital symptoms and/or anogenital ulcers rather than specific laboratory diagnoses. The current national guidelines do not cater for the testing and diagnosis of asymptomatic infections and there are no specific recommendations for the management of extragenital infections [5]. As a result, asymptomatic STI infections in MSM often go undetected and untreated, leading to prolonged illness in infected individuals and continued transmission to sexual partners. The Southern African HIV Clinicians Society (SAHCS) recommends that anorectal and pharyngeal specimens for *Neisseria gonorrhoeae* (NG) testing be considered in MSM [6]. Additionally, the National Department of Health (NDoH) is currently developing guidelines for the management of extragenital sites among key populations, including MSM and transgender women (TGW).

Gonorrhoea and *Chlamydia trachomatis* (CT) infection are among the most prevalent bacterial STIs in MSM globally [7]. A systematic review and meta-analysis on gonococcal and chlamydial prevalence among MSM in sub-Saharan Africa (SSA) reported a pooled prevalence of 27%, with South Africa having the highest burden in the region [8]. Prevalence rates for these pathogens vary by anatomical site, with rectal gonorrhoea globally ranging from 0.2% to 24%, and pharyngeal gonorrhoea from 0.5% to 16.5% [9]. Importantly, pharyngeal gonorrhoea serves as a potential reservoir for emergence of antimicrobial resistance (AMR), emphasising the importance of ongoing surveillance to detect AMR patterns early and guide treatment strategies [10,11]. Notably, South Africa is one of the sentinel countries in the World Health Organization (WHO) Enhanced Gonococcal Antimicrobial Surveillance Programme (EGASP), examining global gonococcal AMR, and together with Thailand the only two WHO EGASP countries that have implemented the WHO EGASP supplementary protocol of extragenital sampling [12,13]. Rectal chlamydia among MSM shows a similar prevalence range as rectal NG, while pharyngeal CT is less common [9]. Although rectal chlamydial infections are usually caused by CT serovars D-K, which are associated with urogenital infections, there is increasing recognition of rectal infections among MSM caused by CT serovars L1 - L3, which can cause lymphogranuloma venereum (LGV). LGV, an invasive and more severe form of chlamydial infection, is frequently detected in MSM practising receptive anal intercourse and can cause complications like lymphadenopathy, rectal ulcers, strictures and fistulas, if untreated [14,15]. Other discharge-causing pathogens, such as *Mycoplasma genitalium* (MG) and *Trichomonas vaginalis* (TV) are less common in MSM, with rectal MG infections more prevalent than urethral and pharyngeal infections, while TV infections are usually reported in less than 1% of cases in MSM [16,17]. Herpes simplex virus type 2 (HSV-2) is currently the leading cause of genital ulceration in South Africa, accounting for 49-60.7% of genital ulcer syndrome (GUS) cases, followed by *Treponema pallidum* subspecies *pallidum* (TP), the causative agent of syphilis, found in up to 26.3% of GUS cases [18,19]. In some regions, HSV-1 has surpassed HSV-2 as the leading cause of genital ulceration. In Australia, HSV-1 now accounts for the majority of genital herpes cases among young MSM and those with anal disease [20]. Given the global rise in HSV-1 as a cause of genital ulceration, it is crucial to monitor its prevalence and impact in South Africa, where data on MSM and other high-risk populations are currently lacking.

This study aimed to (i) estimate the prevalence of urogenital and extragenital STIs among MSM with and without urethral discharge syndrome, (ii) assess the extent of asymptomatic and extragenital infections that would be missed by syndromic management, and (iii) describe phenotypic antimicrobial susceptibility patterns of NG in this population. To achieve these objectives, men with symptoms of urethritis were tested at the urethra, oropharynx, and rectum, while men without urethral symptoms were tested only at extragenital sites. Patients with genital ulcers were also tested for GUS-associated pathogens. Additionally, the study examined phenotypic AMR in NG, providing valuable insights for public health strategies to address STI prevention and treatment in this high-risk population.

## Materials and methods

### Ethics statement

Ethical approval for the present study was granted by the University of the Witwatersrand Human Research Ethics Committee (Medical) (clearance number M210642). All participants provided written informed consent for study participation and use of their data, and indicated whether they agreed to have their samples stored for future STI research. Data were collected on password-protected tablets, and access to the merged dataset was restricted to the data manager, epidemiologist and principal investigator.

### Study design, population and data collection

The study used a cross-sectional design to investigate the aetiologies of STIs among MSM attending the Engage Men’s Health (EMH) Clinic in Rosebank, Johannesburg, South Africa from 24 January to 11 December 2024. EMH is a non-governmental organisation (NGO) clinic dedicated to providing sexual health services and support to MSM, focusing on HIV/STI prevention, testing and treatment. Eligible participants were adult men or transgender women (≥ 18 years) who reported oral or anal sex with another man in the preceding 3 months. MSM presenting with urethral discharge syndrome [21] were enrolled irrespective of any risk factors for STIs in the past 3 months and MSM without urethral discharge syndrome at enrolment were required to self-report at least one risk factor. This approach, aligned with the study’s surveillance objectives and available resources, was intended to maximise the yield of extragenital infections rather than to generate population-level prevalence estimates. Risk factors for STI included ≥5 male partners in the past year, an STI diagnosis in the past 6 months, sex under the influence of drugs in the past 3 months, crystal methamphetamine use in the past 3 months, transactional sex in the past 3 months, sex at a sex-on-premises venue in the past 3 months, sex with an internet- or dating-app sought partner in the past 3 months, accessing HIV PrEP in the past 3 months and condomless receptive anal sex with a casual partner in the past 3 months. After providing written informed consent, participants completed a nurse-administered electronic questionnaire (REDCap) on their demographic, behavioural and clinical characteristics.

### Specimen collection

Oropharyngeal and anorectal specimens were nurse-collected from all participants using ESwab® (Copan Italia SpA, Brescia, Italy). Endo-urethral ESwab® specimens were only obtained from participants with visible urethral discharge. A Dacron ulcer swab was collected from participants presenting with genital and/or peri-anal ulcers. Additionally, a 10 ml venous blood specimen was collected from each participant for HIV, hepatitis B and syphilis serological testing. Clinical specimens were associated with their corresponding questionnaires using unique survey numbers, ensuring no patient-identifying information was included. Swab specimens and blood were transported on ice to the STI Reference Laboratory at NICD within 24 hours. All patients received care in accordance with standard practices at EMH, which included antimicrobial treatment based on South Africa’s national syndromic management guidelines [5].

### Molecular testing

Total nucleic acid was extracted from all swab specimens using the QIAcube HT instrument (Qiagen, Hilden, Germany) following the manufacturer’s instructions. A validated in-house real-time multiplex PCR (mPCR) for urethral/vaginal discharge-causing pathogens was used for the qualitative detection of NG, CT, TV and MG on either the Rotorgene Q (Qiagen, Hilden, Germany) or the Quantstudio 5 (Thermo Fisher Scientific, Waltham, Massachusetts, USA) real-time PCR instruments [22]. Ulcer swab specimens were tested for HSV-1/2, HD, TP and CT using a validated in-house real-time multiplex PCR [23]. All positive results were confirmed using commercial STI assays (Sacace Biotechnologies, Como, Italy). Ulcer and rectal CT-positive samples were screened for LGV (CT serovars L1-L3) using an in-house validated LGV real-time PCR and results were confirmed with a commercial Viasure *C. trachomatis* (LGV) Real Time PCR Detection Kit (CerTest Biotec, Zaragoza, Spain) [24].

### Serological testing

Serum specimens were screened for HIV using the HIV Ag/Ab Combo kit (Abbott Laboratories, Chicago, Illinois, USA) and for active hepatitis B virus (HBV) infection with the HBsAg Next Qualitative assay (Abbott Laboratories, Chicago, Illinois, USA). Syphilis screening was performed according to the reverse algorithm, in which treponemal specific antibodies were detected using the Syphilis TP kit (Abbott Laboratories, Chicago, Illinois, USA) with reactive specimens reflexively tested with a rapid plasma reagin (RPR) assay (BD Macro Vue RPR Card test, Becton Dickinson, Franklin Lakes, New Jersey, USA). All Abbott assays were performed using the ARCHITECT i1000SR analyser (ABBOTT Laboratories, Chicago, Illinois, USA). We analysed RPR titres ≥1:32 separately, as these are indicative of early or active syphilis infection and are associated with higher treponemal burden and increased risk of transmission [25].

### NG culture and antimicrobial susceptibility testing

After vortexing for 30 seconds, a 100 μL volume of each liquid Amies medium containing an Eswab® specimen was inoculated onto a New York City agar plate [26]. Plates were incubated at 35°C-37°C in a 5% CO_2_-enriched atmosphere and examined daily for up to 72 hours. Suspected NG colonies were presumptively identified by Gram-stained microscopy, oxidase and superoxol tests. Additionally, isolates from extragenital sites were identified to species level by matrix-assisted laser desorption ionization with time-of-flight mass spectrometry (MALDI-TOF MS). Antimicrobial susceptibility for cefixime, ceftriaxone, azithromycin and gentamicin was assessed using Etest (bioMérieux, Marcy l’*Etoile*, France), with minimum inhibitory concentration (MIC) values for cefixime, ceftriaxone, and azithromycin interpreted according to European Committee on Antimicrobial Susceptibility Testing (EUCAST) criteria [27]. No EUCAST criteria for gentamicin susceptibility are stated. Notably, due to the high level of resistance, ciprofloxacin is no longer examined during antimicrobial susceptibility testing in South Africa [28]. 2024 WHO NG reference strains were included for quality control [29].

### Data analysis

Demographic, behavioural and clinical data collected during the study period were exported from REDCap into Stata^®^ [version 18.0, Stata Corporation, College Station, Texas] for analysis. Results of the molecular and serological testing for STIs in the same period were extracted from the laboratory information system as Excel^®^ [Microsoft Corporation, Seattle Washington] sheets and exported into Stata^®^ for analysis. After data cleaning and consistency checks, the demographic, behavioural and clinical data were merged with laboratory data for analysis. Descriptive statistics were used to characterise the enrolled participants and to estimate the prevalence of urogenital and extragenital STIs, with comparisons made between individuals with and without urethritis. For NG antimicrobial susceptibility testing, the analysis included determining the MIC range, MIC_50_ and MIC_90_ (the concentrations required to inhibit 50% and 90% of isolates, respectively) and the proportion of isolates, which were susceptible to ceftriaxone, cefixime, and azithromycin based on EUCAST criteria [23]. Statistical differences between groups were assessed using Chi-squared test, Fisher`s exact test and Wilcoxon rank-sum test, as appropriate. A p-value < 0.05 was considered statistically significant, and 95% confidence intervals (CIs) were calculated where applicable. Logistic regression was used to examine associations between selected demographic and behavioural factors and specific STI outcomes. Factors associated with an outcome at p < 0.20 in initial analyses were considered for inclusion in multivariable models. Separate models were developed for participants with and without urethral discharge syndrome due to differences in clinical presentation and outcome frequency. Final models were constructed using a stepwise approach, retaining variables that remained statistically significant (p < 0.05) and ensuring there was not more than one variable per 10 outcomes in each final model. Because some STI outcomes were uncommon, we limited the number of variables included in each model to one variable per outcome to improve the stability of the estimates. For the purposes of this analysis, ‘Any STI’ was defined as the presence of any urethral discharge-causing STI, including NG, CT, TV and MG.

## Results

### Demographic, behavioural and clinical characteristics of participants

The demographic, behavioural and clinical factors of the participants are presented in Table 1. Among the 189 MSM recruited in 2024 (128 without urethral symptoms and 61 with urethritis), the two groups were demographically similar, with comparable median ages (30 vs 29 years), income distribution and ethnic composition, the majority being African (68.8%). Most participants identified as male (99.5%) and homosexual (65.6%), with 32.3% identifying as bisexual. Across the cohort, high-risk sexual behaviours were common: 70.9% reported ≥5 male partners in the past year, one in five had been diagnosed with an STI in the preceding 6 months or had sex under the influence of drugs in the past 3 months, 65.1% had sex with an internet-sought partner and nearly half had accessed HIV PrEP. However, several significant differences were observed between MSM with and without urethritis. Those without urethral symptoms were more likely to have sex exclusively with men (82.8% vs 65.6%, p = 0.008), engage in condomless receptive anal sex with a casual partner (70.3% vs 39.3%, p < 0.001) and report having a partner with urethral discharge (46.1% vs 1.6%, p = 0.001). Rectal symptoms were also concentrated in this group, with 17.2% reporting rectal pain (vs 0%, p < 0.001) and 7.8% reported pain during defecation (vs 0%, p = 0.032). Knowledge of HIV status was high (99%), with approximately one in three MSM self-disclosing as living with HIV (31.6%).

**Table 1:** Demographic, behavioural and clinical factors in MSM with and without urethritis.

	Presence of Urethritis Symptoms			
Variable, n (%)	No	Yes	All	p-value^Ξ^
	(N = 128)	(N = 61)	(N = 189)	
**Demographic Factors**				
Age, median (IQR) in years	30 (26 - 36)	29 (25 – 34)	30 (26 - 36)	0.365 ^*W*^
Ethnic group				
African	86 (67.2)	44 (72.1)	130 (68.8)	
Mixed race	6 (4.7)	0 (0.0)	6 (3.2)	0.329 ^**Ξ**^
Indian	3 (2.3)	0 (0.0)	3 (1.6)	
White	32 (25.0)	17 (27.9)	49 (25.9)	
Gender Identity				
Male/Man	128 (100.0)	60 (98.4)	188 (99.5)	0.323 ^**Ξ**^
Non-binary	0 (0.0)	1 (1.6)	1 (0.5)	
Sexual orientation (sex with)				
Homosexual (men only)	89 (69.5)	35 (57.4)	124 (65.6)	0.204 ^**Ξ**^
Bisexual (men and women)	36 (28.1)	25 (41.0)	61 (32.3)	
Pansexual (any gender identity)	3 (2.3)	1 (1.6)	4 (2.1)	
Monthly income (in ZAR)				
<2,500	21 (16.4)	10 (16.4)	31 (16.4)	0.346 ^**Ξ**^
2,500- 5,000	17 (13.3)	4 (6.6)	21 (11.1)	
5,001- 10,000	22 (17.2)	16 (26.2)	38 (20.1)	
>10,000	68 (53.1)	31 (50.8)	99 (52.4)	
**Behavioural Factors**				
Sex partner type in the past 3 months				
Men only	106 (82.8)	40 (65.6)	146 (77.3)	**0.008**
Men and women	22 (17.2)	21 (34.4)	43 (22.8)	
Number of sex partners in the past 3 months (median, IQR)				
Regular sex partners	1 (0 -2)	1 (1 -2)	1 (0- 2)	0.502 ^*W*^
Casual sex partners	3 (2-4)	3 (1-5)	3 (1 –5)	0.970 ^*W*^
In the past 12 months, had ≥ 5 sex partners	91 (71.1)	43 (70.5)	134 (70.9)	0.932
In the past 6 months, had an STI diagnosis	29 (22.7)	12 (19.7)	41 (21.7)	0.642
In the past 3 months				
Exchanged in transactional sex for money/goods	2 (1.6)	0 (0.0)	2 (1.1)	1.000 ^**Ξ**^
Exchanged in sex at SOPV	39 (30.5)	20 (32.8)	59 (31.2)	0.748
Exchanged in tongue kissing	127 (99.2)	61 (100.0)	188 (99.5)	1.000 ^**Ξ**^
Had sex with an internet/app-sought partner	81 (63.3)	42 (68.9)	123 (65.1)	0.453
Had sex under the influence of drugs	27 (21.1)	9 (14.8)	36 (19.1)	0.299
Had condomless RAI with a casual partner	90 (70.3)	24 (39.3)	114 (60.3)	**<0.001**
Had condomless receptive oro-penile sex (fellatio)	103 (80.5)	42 (68.9)	145 (76.7)	0.077
Had insertive oro-anal sex (rimming)	70 (54.7)	33 (54.1)	103 (54.5)	0.939
Had receptive oro-anal sex (rimming)	79 (61.7)	32 (52.5)	111 (58.7)	0.227
Had sex with a partner living in another SA province	24 (18.8)	11 (18.0)	35 (18.5)	0.906
Had sex with a partner living in another country	25 (19.5)	8 (13.1)	33 (17.5)	0.277
Accessed HIV PrEP	62 (48.44)	30 (49.2)	92 (48.7)	0.924
Partner/s have urethral discharge				
Yes	59 (46.1)	1 (1.6)	12 (6.4)	**0.001**
No	58 (45.3)	12 (19.7)	71 (37.6)	
Do not know	11 (8.6)	48 (78.7)	106 (56.1)	
Willing to refer sex partners for treatment	73 (57.0)	32 (52.5)	105 (55.6)	0.554
**Clinical Factors**				
Ever circumcised	67 (52.3)	36 (59.0)	103 (54.5)	0.389
Medical Circumcision	55/67 (82.1)	28 (77.8)	83 (80.6)	0.598
Traditional Circumcision	12/67 (17.9)	8 (22.2)	20 (19.4)	
Knowledge of HIV status	127 (99.2)	60 (98.4)	187 (98.9)	0.542 ^**Ξ**^
Self-reported as living with HIV	44/127 (34.7)	15/60 (25.0)	59/187 (31.6)	0.185
Self-reported as not living with HIV	83/127 (65.4)	45/60 (75.0)	128/187 (68.5)	
Self-reported ART use in the past 3 days	38 (97.4)	13 (100.0)	51 (98.1)	1.000 ^**Ξ**^
Presented with the following symptoms at enrolment				
Sore throat	5 (3.9)	0 (0.0)	5 (2.7)	0.177 ^**Ξ**^
Testicular tenderness	2 (1.6)	0 (0.0)	2 (1.1)	0.458 ^**Ξ**^
Rectal Discharge	6 (4.7)	0 (0.0)	6 (3.2)	0.093 ^**Ξ**^
Rectal Bleeding	3 (2.3)	0 (0.0)	3 (1.6)	0.056 ^**Ξ**^
Rectal Pain	22 (17.2)	0 (0.0)	22 (11.6)	**<0.001** ^**Ξ**^
Pain during defecation	10 (7.8)	0 (0.0)	10 (5.3)	**0.032** ^**Ξ**^
Used Antibiotics in the past 2 weeks	3 (2.3)	2 (3.3)	5 (2.7)	0.658 ^**Ξ**^

Abbreviations: ART: HIV antiretroviral therapy, RAI: receptive anal intercourse, SOPV: sex on site premises, PrEP: HIV pre-exposure prophylaxis, ZAR: the South African Rand. Ξ Fisher’s Exact test, unless otherwise indicated, W Rank Sum Test

### Prevalence of STI pathogens by anatomical site

**Fig 1:**
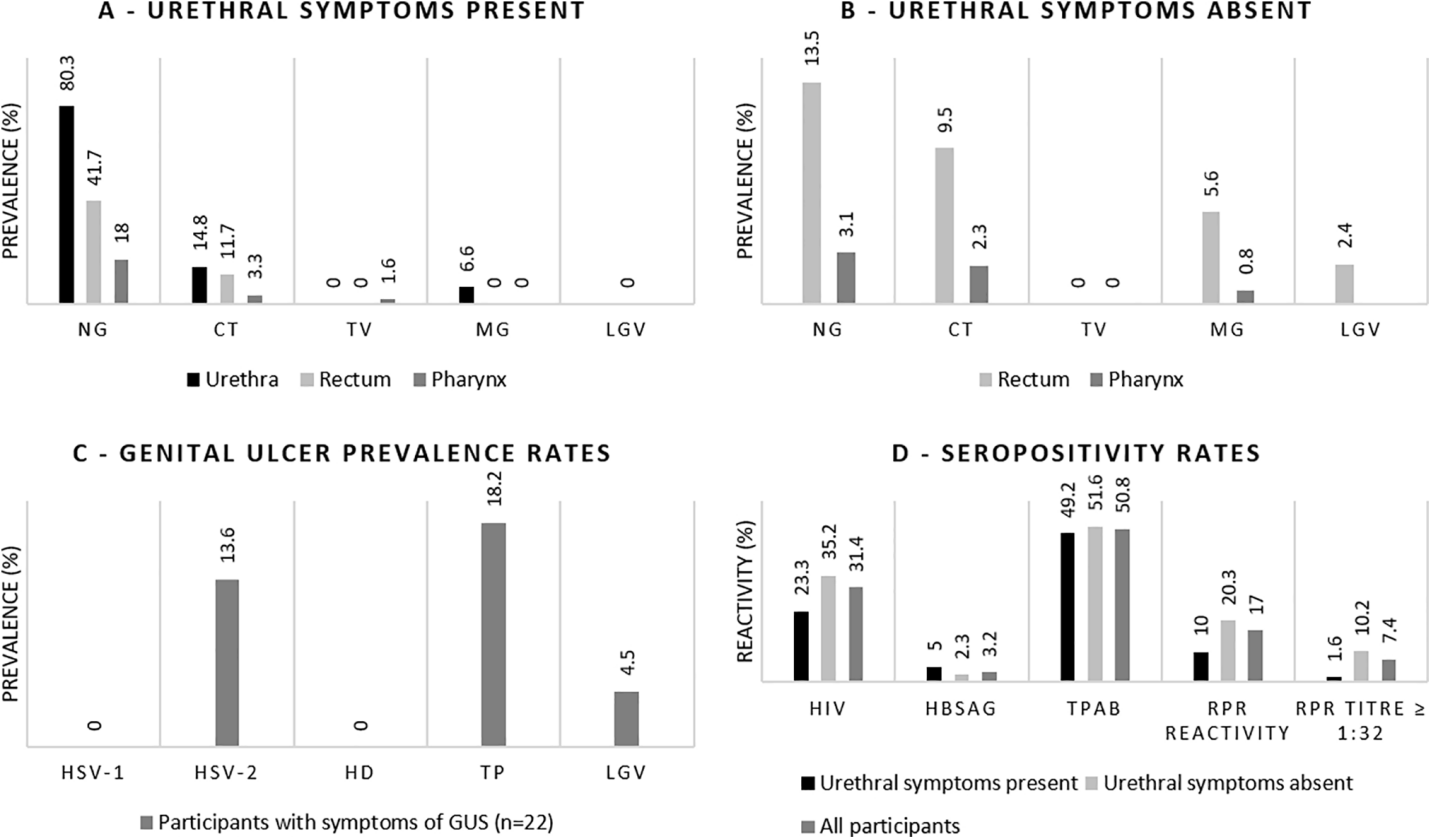
A: Site-specific prevalence of STIs among participants with urethral symptoms (n = 61); B: participants without urethral symptoms (n = 126); C: distribution of STI pathogens among participants with genital ulcer syndrome (n = 22) and D: serological findings by urethritis status. Abbreviations: NG: *Neisseria gonorrhoeae*, CT: *Chlamydia trachomatis*, TV: *Trichomonas vaginalis*, MG: *Mycoplasma genitalium*, LGV: lymphogranuloma venereum, HSV-1: Herpes simplex virus type 1, HSV-2: Herpes simplex virus type 2, HD: *Haemophilus ducreyi*, TP: *Treponema pallidum*, HIV: human immunodeficiency virus, HBSAG: Hepatitis B surface antigen, RPR: rapid plasma reagin.

All urethral and oropharyngeal samples produced evaluable results, whilst three rectal samples yielded invalid results and were excluded from the analysis. Figs 1A and 1B summarise STI prevalence at urethral, rectal and oropharyngeal sites among MSM with and without urethral symptoms, respectively. Among men with urethritis*,* NG was the most prevalent pathogen, detected in 80.3% (49/61) of urethral, 41.7% (25/60) of rectal, and 18.0% (11/61) of oropharyngeal samples. Concurrent NG infections across sites were common: 49.0% (24/49) of MSM with urethral NG were also infected with NG rectally, whilst 20.4% (10/49) were also infected with NG at the oropharynx (data not shown). CT prevalence was lower than that of NG, detected in 14.8% (9/61) of urethral, 11.7% (7/60) of rectal and 3.3% (2/61) of oropharyngeal samples, respectively. MG was identified only in four urethral samples (6.6%, 4/61), while TV was detected only in one oropharyngeal sample (1.6%, 1/61). Urethral co-infections, with NG/CT, NG/MG and CT/MG, were detected in 9.8%, 3.3% and 1.6% of participants, respectively. Among men without urethritis, for which urethral testing was not performed, NG was detected in 13.5% (17/126) of rectal and 3.1% (4/128) of oropharyngeal samples and was significantly less prevalent at both extragenital sites compared to MSM with urethral symptoms (rectal p < 0.001; oropharyngeal p = 0.001). CT was detected in 9.5% (12/126) of rectal and 2.3% (3/128) of oropharyngeal samples and the prevalence did not differ from those with urethritis. MG was detected in 5.6% (7/126) of rectal and 0.8% (1/128) of oropharyngeal samples, while no TV infections were identified. Among participants without urethritis, 18/128 (14.1%) of NG, 15/128 (11.7%) of CT, and 8/128 (6.3%) of MG infections at extragenital sites would have been missed under syndromic testing alone. Across the entire cohort, 43/187 (23.0%) participants had ≥ 2 concurrent infections across anatomical sites and/or pathogens (Table 2). The predominant combination involved dual-site NG infection, most commonly urethral and rectal NG (17 cases). NG was detected at three anatomical sites in 4 participants. Additional multi-site and multi-pathogen combinations are described in Table 2. Among rectal CT infections, all cases in participants with urethritis were LGV-negative, whereas 3/12 (25.0%) in those without urethritis were LGV-positive, corresponding to 3/126 (2.4%) of all participants without urethritis. Overall, 3/186 (1.6%) participants were LGV-positive. Genital ulcers were observed in 22 participants without urethritis (Fig 1C); of these, 3/22 (13.6%) tested positive for HSV-2, 4/22 (18.2%) for TP and 1/22 (4.5%) for LGV. Of the four participants who tested positive for LGV (3 rectal and 1 genital ulcer), two were also positive for HIV (2/4; 50%).

**Table 2:** Individual-level concurrent infections across anatomical sites (N = 187).

Infection combination	n (%)
Urethral NG + Rectal NG	17 (9.1)
Urethral NG + Pharyngeal NG	3 (1.6)
Rectal NG + Pharyngeal NG	2 (1.1)
Urethral NG + Rectal NG + Pharyngeal NG	4 (2.1)
Urethral NG + Urethral CT	5 (2.7)
Rectal NG + Rectal CT	3 (1.6)
Rectal NG + Rectal MG	1 (0.5)
Urethral NG + Urethral MG + Rectal CT	1 (0.5)
Urethral CT + Urethral MG + Rectal CT	1 (0.5)
Rectal NG + Rectal CT + Pharyngeal NG	1 (0.5)
Pharyngeal NG + Rectal NG + Rectal CT + Urethral NG	1 (0.5)
Urethral NG + Rectal NG + Rectal CT + Pharyngeal CT	1 (0.5)
Urethral NG + Rectal NG + Rectal CT + Pharyngeal NG + Pharyngeal CT	1 (0.5)
Urethral NG + Urethral CT + Pharyngeal NG	1 (0.5)
Rectal CT + Pharyngeal TV	1 (0.5)

Each row represents one individual infection; categories are non-overlapping; NG, *Neisseria gonorrhoeae*; CT, *Chlamydia trachomatis*; MG, *Mycoplasma genitalium*; TV, *Trichomonas vaginalis*

### Serological results

The seroprevalence of HIV, HBsAg and treponemal antibodies were 31.4%, 3.2%, 50.8%, respectively (Fig 1D). Among participants with detectable TP antibodies, 32/95 (33.7%) were RPR reactive. Overall, serological findings did not differ significantly between participants with and without urethritis, although high RPR titres (≥ 1:32) were less frequently observed in the urethritis group (p = 0.039). Overall, approximately one in six MSM were RPR reactive, including one in ten with urethritis and one in five without urethral symptoms.

### NG MIC values for antibiotics across different anatomical sites

The isolation rates for NG varied across the different anatomical sites. From the urethra, NG was successfully cultured in 39/49 (79.6%) samples that were NG positive in the mPCR. In rectal samples, NG isolation occurred in 14/42 (33.3%) NG-positive PCR samples. The lowest isolation rate was observed in the oropharyngeal samples, with NG being isolated in 3/15 (20.0%) NG-positive PCR samples. The NG MIC results for all antibiotics across different anatomical sites (urethral, rectal and oropharyngeal) showed no resistance in the isolates tested (Table 3). For all three sites, ceftriaxone, cefixime, and azithromycin exhibited 100% susceptibility, and the MICs of gentamicin were low.

**Table 3:** NG MIC values for antibiotics across different anatomical sites (urethral, rectal and pharyngeal).

Site of sample collection	Antibiotic	Number of NG isolates	Minimum MIC	MIC_50_	MIC_90_	Maximum MIC
Urethral	Ceftriaxone	39	< 0.002	0.002	0.008	0.016
	Cefixime		< 0.016	< 0.016	0.016	0.064
	Gentamicin		0.5	2	4	4
	Azithromycin		0.016	0.125	0.5	1
Rectal	Ceftriaxone	14	< 0.002	0.004	0.008	0.008
	Cefixime		< 0.016	< 0.016	0.032	0.064
	Gentamicin		1	2	4	4
	Azithromycin		0.016	0.125	0.25	0.25
Pharyngeal	Ceftriaxone	3	< 0.002	0.002	0.016	0.016
	Cefixime		< 0.016	< 0.016	0.064	0.064
	Gentamicin		1	2	2	2
	Azithromycin		0.016	0.064	1	1

MIC: minimum inhibitory concentration in mg/L. Breakpoint criteria as per EUCAST criteria for 2025 [23]. Azithromycin: Susceptible ≤ 1 mg/L (ECOFF); Cefixime: Susceptible ≤ 0.125 mg/L; Ceftriaxone: Susceptible ≤ 0.125 mg/L

### Factors associated with STIs among MSM according to the presence/absence of urethral symptoms

Risk factors associated with STIs among MSM (at any anatomical site) according to the presence/absence of urethral symptoms are summarised in supplementary tables (S1 Table and S2 Table). Participants with symptoms of urethritis who practiced insertive oro-anal sex in the past three months were 82% less likely to be infected with CT [aOR 0.18 (95% CI 0.05-0.71), p = 0.014], while those who accessed HIV PrEP in the last three months were 77% less likely to be infected with CT [aOR 0.23 (95% CI 0.06- 0.92), p = 0.038]. No other factors in the final multivariable model were associated with having NG, CT or any discharge STI in participants with urethral symptoms. Among those without urethral symptoms, homosexual orientation [aOR 3.09 (95% CI 1.11- 8.65), p = 0.031], not being medically circumcised [aOR 2.97 (95% CI 1.19-7.44), p = 0.012] and being HIV positive [aOR 4.03 (95% CI 1.71-9.50), p = 0.001] significantly increased the risk of having an STI. Specifically, HIV-positive individuals [aOR 3.39 (95% CI 1.17-9.87), p = 0.025] and those who were not medically circumcised [aOR 7.23 (95% CI 1.56-33.51), p = 0.011] had a higher likelihood of testing NG positive. Participants without urethral symptoms who were HIV positive had a higher likelihood of being infected with CT [aOR 3.42 (95% CI 1.10- 10.59), p = 0.033].

## Discussion

We investigated the prevalence of STI pathogens and AMR in NG at urogenital and extragenital sites among MSM attending the EMH Clinic in Rosebank, Johannesburg, South Africa in 2024, stratified by urethral discharge syndrome status. The findings of the study represent sentinel surveillance data from a specialised NGO clinic serving MSM in urban Johannesburg. Participants were recruited from a setting characterised by high STI risk and care-seeking behaviour, and therefore prevalence estimates may not reflect the broader MSM population. The study showed a high burden of STIs among MSM presenting with urethral discharge, at the urethra (53/61; 86.9%), but also at extragenital sites (33/61; 54.1%). Similarly, extragenital STI infections were observed in 28.6% (36/126) of MSM without any urethral symptoms, highlighting the importance of extragenital sites as a potential reservoir for STI transmission and the need for multi-site screening in MSM populations. Urethral specimens were not collected from participants without urethral symptoms, which likely resulted in an underestimation of urethral STI prevalence in asymptomatic MSM. This limitation also affects comparisons between symptomatic and asymptomatic groups, as some infections classified as ‘extragenital only’ may have been accompanied by undetected urethral infection. However, this approach reflects current syndromic management practice in South Africa and highlights the extent to which urethral and extragenital infections would be missed under symptom-based testing strategies.

Culture recovery of NG was lower than PCR detection rates, particularly for extragenital samples. This is expected, as extragenital infections typically have lower bacterial loads and are more susceptible to overgrowth by commensal flora, reducing culture sensitivity. In addition, the fastidious nature of NG and delays between sampling and culture may have further compromised culture yield. Despite these limitations, all recovered isolates across anatomical sites demonstrated no resistance to ceftriaxone, cefixime, and azithromycin, with low MICs for gentamicin. The study also highlighted the substantial burden of HIV and syphilis in this cohort, with over one-third of participants testing HIV-positive and a similar proportion presenting with recent or active syphilis infection. Self-reported HIV status closely matched actual testing results (31.6% vs. 31.4%), suggesting that participants’ reports were generally reliable in this cohort. These findings emphasise the need for integrated HIV and syphilis prevention and treatment strategies, alongside STI screening, which are tailored to MSM.

Urethral NG prevalence in our symptomatic MSM cohort was similar to rates (83.1 – 87.8%) reported among heterosexual men with male urethritis syndrome (MUS) at South African STI surveillance sites (2019–2024) [30,31]. Our findings are broadly consistent with international literature demonstrating a substantial burden of extragenital NG and CT infections among MSM. However, prevalence estimates vary considerably across settings due to differences in study populations, sampling strategies, and testing practices [9]. As our study utilised a convenience sample of clinic-attending MSM, the prevalence estimates are not population-representative and should be interpreted as reflecting the burden among healthcare-seeking individuals rather than the wider MSM population. Notably, consistent with global reports, a substantial proportion of extragenital infections were asymptomatic and would have been missed with urogenital screening alone. Although South African data on extragenital STI infections in MSM are limited, a study by Rebe *et al*. (2015) involving 200 MSM in Cape Town, reported that the majority of NG/CT infections were asymptomatic and extragenital, with rectal and pharyngeal sites more frequently affected than the urethra [32]. A more recent study by Da Costa Dias *et al* (2024) reported extragenital infections in 24.7% of asymptomatic MSM in Johannesburg during 2022, with rectal NG and CT prevalence rates comparable to those observed in our study [33]. While the Da Costa Dias *et al* (2024) study reported only NG infections at the pharyngeal site, our study also identified pharyngeal CT infections, though at a low prevalence (2.3%). Pharyngeal CT is uncommon among MSM, clears spontaneously and is not a major contributor in CT transmission at population level [34]. LGV was detected in three asymptomatic participants with rectal CT (3/12; 25.0%) and in one additional asymptomatic participant who presented with a genital ulcer (1/22; 4.5%). Two of the four LGV-positive individuals in our cohort were living with HIV, but none of the rectal LGV-positive participants reported rectal pain or pain during defecation. These findings align with global reports indicating that a large proportion of LGV infections among MSM are asymptomatic and that it is more frequently detected among HIV-positive individuals [35,36]. LGV infections have more serious long-term health consequences and require an extended course of treatment, but these infections can only be detected through aetiological testing. This highlights the need for robust surveillance systems across diverse populations to improve the diagnosis and effective treatment of LGV infections [37].

Pharyngeal MG was detected in only one participant (0.8%) and rectal MG in seven participants (5.6%), all of whom were asymptomatic. These findings are consistent with previous studies, which found that MG is not a frequent coloniser of the pharynx but is more prevalent at the rectal site [16]. However, the clinical significance of MG infections at extragenital sites is unclear, especially in asymptomatic individuals [16,38]. Routine treatment of MG infections at extragenital sites is not currently recommended due to limited evidence of pathogenicity and rising macrolide and fluoroquinolone resistance [39]. No pharyngeal or rectal TV infections were identified in our asymptomatic MSM cohort, supporting previous reports that TV is also not common at extragenital sites. Pharyngeal TV infection was detected in only one participant who presented with symptoms of urethritis, despite no STI being detected in the urethra. This rare finding may reflect limited or infrequent oral exposure to TV-infected partners and is likely transient [40,41]. Misclassification with the oral commensal *Trichomonas tenax* is also unlikely, as detection was performed using a species-specific multiplex PCR and confirmed with a commercial TV-specific PCR assay. Nearly a quarter of participants (23.0%) had two or more concurrent infections, highlighting the need for multisite screening in clinical practice. Undetected coinfections can lead to suboptimal treatment, persistent infection, and ongoing transmission. Clinicians should consider comprehensive testing strategies, particularly in high-risk populations such as MSM, to ensure appropriate therapy and reduce onward spread.

Genital ulcer aetiology in our asymptomatic cohort was mainly due to infection with TP and HSV-2 (18.2% and 13.6% of ulcer cases, respectively). Notably, HSV-1 was not identified as a cause of genital ulceration in our cohort. This finding aligns with studies from sub-Saharan Africa, where HSV-2 remains the predominant HSV type associated with GUS, and where HSV-1 is less frequently associated with primary genital infections [18,42]. The observed seroprevalence of HIV (31.4%), HBsAg (3.2%) and previous syphilis exposure (50.8%) reflects a high burden of STIs in our MSM population. Among those seropositive for TP, 33.7% were RPR-positive, indicating recent or active infection. These seroprevalence rates align with other high‐risk cohorts in Southern Africa, which are disproportionately affected by HIV and where syphilis exposure rates often exceed 20–40% [43–46]. This indicates the need to expand serological screening for syphilis beyond antenatal and HIV care settings to other high-risk populations, such as MSM.

Several factors were associated with STI diagnosis among study participants. Among MSM with urethritis, those who had accessed HIV PrEP in the past three months had approximately 4.3-fold lower odds of CT infection, which could be due to more frequent STI screening, prompt treatment and engaging in safer sexual practices rather than a direct effect of PrEP itself. Recent insertive oro-anal sex (rimming) was associated with more than fivefold lower odds of CT infection. The inverse association with rimming and CT infection should be interpreted cautiously given the small sample size, potential residual confounding, and multiple comparisons. The observed finding may be misleading due to these factors rather than reflecting a true protective association. These were the only factors that remained significant in multivariable analysis. Among asymptomatic MSM, independent risk factors for STI acquisition included homosexual orientation, lack of medical circumcision and HIV-positive status. The association between HIV and increased STI risk is consistent with prior evidence linking immunosuppression, high-risk networks and overlapping sexual practices to co-infections [47,48]. Similarly, while medical circumcision has been shown to provide partial protection against HIV in MSM, evidence for its effect on other STIs remains mixed [49,50]. These findings emphasise the need to prioritise routine extragenital STI screening for asymptomatic MSM, particularly those who are HIV-positive or uncircumcised. Most of the behavioural factors in this study were not associated with STI risk, whereas biological factors such as HIV positivity and circumcision status showed stronger associations. This suggests that relying on behavioural risk assessments to determine who should be tested is suboptimal. Instead, routine STI screening is essential, as risk-based testing approaches have been shown to be ineffective [51].

In our study, all NG isolates across urethral, rectal and pharyngeal sites were fully susceptible to ceftriaxone, cefixime, and azithromycin, and had low MICs of gentamicin. Although no resistance was detected among the NG isolates tested, particularly from extragenital sites, the small number of rectal and pharyngeal isolates limits definitive conclusions regarding susceptibility patterns at these sites. This contrasts with recent heterosexual STI surveillance in South Africa, where the emergence of high-level azithromycin-resistant NG has been documented [31,52]. While our sample size is relatively small, AST data for MSM from South Africa remain scarce, representing a key strength of this study. Furthermore, sequencing efforts to assess molecular determinants of AMR in these NG isolates, as well as for other STI pathogens such as MG, CT and TP, are currently underway. These analyses will enable comparison with global strains and offer important insights into how AMR emerges and spreads within MSM populations.

This study has several limitations. First, due to the cross-sectional design, we cannot determine whether the reported behaviours preceded STI acquisition. Second, urethral testing was not performed in asymptomatic participants, likely underestimating the true burden of urethral infections in this cohort. Third, asymptomatic participants were required to report at least one recent STI risk factor, resulting in different selection criteria for symptomatic and asymptomatic groups. Consequently, prevalence estimates among asymptomatic MSM may not reflect the broader clinic population, and direct comparisons between groups should be interpreted with caution. Fourth, self-reported sexual behaviours are subject to recall and social desirability biases, which may have impacted the accuracy of our risk factor analysis. Fifth, the generalisability of our findings is limited, as participants were recruited from a specialised NGO clinic in an urban setting and may not reflect MSM in other settings. Sixth, several subgroup and multivariable analyses were based on small numbers, particularly for LGV infections and analyses stratified by urethral symptom status. Some adjusted estimates were unstable and characterised by large effect sizes with wide confidence intervals and should therefore be interpreted with caution due to limited statistical power. Finally, the number of NG isolates from pharyngeal and rectal sites was small, limiting our ability to draw definitive conclusions regarding antimicrobial susceptibility patterns at these anatomical sites.

Our findings demonstrate a high burden of asymptomatic and extragenital STIs among MSM in Johannesburg, particularly among HIV-positive and uncircumcised MSM, highlighting the limitations of South Africa’s current syndromic management approach [5]. These data reinforce the recommendation of the SAHCS to consider anorectal and pharyngeal testing for NG in MSM and provide local evidence to inform the ongoing development of extragenital management guidelines by the NDoH. In this context, our findings support the implementation of routine, anatomical site-specific molecular STI screening for MSM, particularly those at higher risk of infection. Incorporating extragenital screening into national guidelines may improve case detection, reduce ongoing transmission, and contribute to AMR surveillance efforts. While the consistent antimicrobial susceptibility observed in NG isolates is reassuring, ongoing AMR surveillance remains essential. Efforts to strengthen partner notification and integrate aetiological STI testing into routine care will be critical. The forthcoming national STI guidelines for key populations present an opportunity to embed these strategies, enhance early detection and treatment and ultimately reduce transmission and improve health outcomes among MSM in South Africa.

## Supporting information

S1 Table Factors associated with STIs among MSM with urethral symptoms. (XLSX)

S2 Table Factors associated with STIs among MSM with no urethral symptoms. (XLSX)
